# Effects of Short-Term Set-Aside Management Practices on Soil Microorganism and Enzyme Activity in China

**DOI:** 10.3390/ijerph14080913

**Published:** 2017-08-14

**Authors:** Guangyu Li, Cifang Wu

**Affiliations:** 1 Institute of Land Science and Property Management, School of Public Affairs, Zhejiang University, Hangzhou 310058, China; sethlee0010@yahoo.com; 2 Land Academy for National Development, Zhejiang University, Hangzhou 310058, China

**Keywords:** set-aside patterns, microbial communities, microbial biomass carbon, farmyard manure, soil enzyme

## Abstract

Set-aside farmland can effectively improve the self-rehabilitation of arable soil. Long-term set-asides however cannot satisfy provisionment, therefore the use of short-term set-asides to restore cultivated soil is a better option. Few studies have compared short-term set-aside patterns, and the effects of set-asides on soil microbial community and enzyme enzymes. We analyzed the bacterial structure, microbial biomass carbon/nitrogen and enzyme activity of farmland soil under different set-aside regimes in the Yellow River Delta of China. Bacterial alpha diversity was relatively lower under only irrigation, and farmyard manure applications showed clear advantages. Set-asides should consider their influence on soil organic carbon and nitrogen, which were correlated with microbial community structure. *Nitrospira* (0.47–1.67%), Acidobacteria *Gp6* (8.26–15.91%) and unclassified Burkholderiales (1.50–2.81%) were significantly altered (*p* < 0.01). Based on functions of these genera, some set-aside patterns led to a relative balance in nitrogen and carbon turnover. Partial treatments showed a deficiency in organic matter. In addition, farmyard manure may lead to the increased consumption of organic matter, with the exception of native plants set-asides. Conventional farming (control group) displayed a significant enzyme activity advantage. Set-aside management practices guided soil microbial communities to different states. Integrated soil microbiota and the content of carbon and nitrogen, native plants with farmyard manure showed an equilibrium state relatively, which would be helpful to improve land quality in the short-term.

## 1. Introduction

According to the Ministry of Land and Resources of the People’s Republic of China, approximately 2.67% of the arable land in China is in excellent condition based on the Chinese standard “Regulation for gradation on agriculture land quality” (GB/T 28407-2012) defining soil fertility, productivity, irrigation, etc. [1]. Increasing amounts of chemical inputs (herbicides and fertilizers) are available to provide stable farm incomes in areas with formerly poor environmental conditions [2]. However, water and soil erosion of arable land results from this behavior, and organic matter quality and quantity becomes degraded. The degradation of organic matter destroys the soil microarea, and threatens microorganism survival [3]. A growing number of scientists, farmers and the general public fear for the long-term sustainability of such highly input-dependent and ecologically simplified food production systems [4]. To improve farmland ecosystems, the European Union (EU) proposed in 1992 a set-aside programme, which is part of the Common Agricultural Policy (CAP) [5]. Regarding long-term set-aside, the Chinese government implemented the Grain for Green programme in 1999 [6]. However, due to increasing human needs for land, restoring large areas of farmland to forest or grassland is unrealistic [7]. Short-term set-aside projects are more suitable for China, but remain immature. 

The relevant studies on set-asides in China have mainly focused on rotation crop, no-tillage, rotation tillage and barely fallow practices. We designed valid set-aside methods according to ecological intensification based on agronomic knowledge, plant science and agricultural management [8,9,10]. Therefore, three types of set-aside methods were established: (i) intercropping and mixed sowing instead of monoculture [11]; (ii) changing the crops on arable land; (iii) improving the management of farmland and relieving the pressure of traditional farming. 

The health of arable land is closely linked to soil microbial properties [12], and the capability of cultivated land is significantly correlated with microbial biomass carbon (MBC) and microbial biomass nitrogen (MBN) [13]. Microbial diversity and enzyme activity can reflect the type of tillage, type of crop, input of nitrogen fertilizer and effect of land reclamation [14,15]. Microbial diversity and enzyme activity have also been used to assess soil quality in contaminated soils [16]; therefore, they could represent important indexes for evaluating the sustainable development of cultivated land. Some scholars have proposed that soil biodiversity is crucial to agro-ecosystems and thus should be included in the scope of management decision-making [17].

Determining the eco-efficiency of set-aside farmland with respect to soil microbiology is necessary. We used a soil microbial method to study the mechanism of different set-aside methods on the microbial community structure and enzyme activity. Our results addressed: (i) the short-term effects of different set-asides on soil microbes and (ii) the choice of a suitable set-aside method.

## 2. Methods

### 2.1. Site Description and Experimental Design

The study site was located at the Shandong Wudi Field Scientific Observation and Research Base for Land Use, in Binzhou, China (E 117°43’, N 37°48’, altitude of 5 m above sea level). The soil was approximately 5% clay and 75% silt; it is derived from diluvial sediments of the Yellow River and is classified as a typical saline alluvial soil (Fluvisols, per the Food and Agriculture Organization). According to the local agricultural bureaus, sorghum has been conventionally planted for 5 years (sown in April, harvested in October and fallow in winter), but the planting area decreased markedly where partial farmland (45%) nearby was already or nearly abandoned, grown with other crops or changed to water areas. The experiments were set up in May 2015 and concluded in May 2016, on flat and relatively homogeneous farmland. 

The topsoil (0–20-cm layer) chemical properties were as follows: electrical conductivity (EC) of 1.8 μS/m, total nitrogen (TN) of 0.62 g/kg, soil organic carbon (SOC) of 8.39 g/kg, available nitrogen (AN) of 24.59 mg/kg, available potassium (AK) of 0.11 mg/kg, available phosphorus (AP) of 8.51 mg/kg, and a pH of 8.43.

We chose soybean (*Glycine max* (Linn.) Merr.), maize (*Zea mays* L.), alfalfa (*Medicago sativa* L.), dahurian wild rye (*Elymus dahuricus* Turcz.) and native plants after combining the opinions of the local agriculture sectors, an investigation of local cultivation customs and farmer preferences and the benefits of both mixed seeds and intercropping of leguminous and gramineous plants [18]. The vegetation types in conjunction with three management regimes formed 15 set-aside patterns and a control treatment; relevant abbreviations are shown in Table 1. Each pattern was replicated three times, for a total of 48 plots (5 × 6 m^2^ each) that were randomly distributed (more than 1 m interval each for a total area of 2.6 ha). Management treatments began 5 May 2015 and continued for 1 year. Treatment details were as follows: (i) no management (N1–N5), in which no other labour except surface weeding occurred to ensure the emergence of plants (N2–N5); (ii) irrigation only (I1–I5), in which irrigation occurred on 13 May 2015 using water from nearby rivers (the irrigation amount was calculated using the ETo calculator version 3.1 (developed by the FAO http://www.fao.org/nr/water/eto.html)) and I2–I5 treatments were surface-weeded; (iii) organic manure and irrigation (O1–O5), in which both cow manure (1500 kg/ha) and irrigation were applied and O2–O5 treatments were surface-weeded; and (iv) control group (CK), in which conventional sorghum planting occurred alongside tilling and simultaneous flood irrigation applications as well as chemical fertilizer (urea and inorganic fertilizer (NPK) for a total of 330 kg/ha). Based on (i) to (iii), we chose the intercropping sowing method to guarantee feasibility.

### 2.2. Soil Sampling and Preparation

The treatments began 5 May 2015. On 10 June 2016, we completed soil sampling. According to the “S” sampling principle, we collected soil samples from six points at a depth of 0–20 cm from each plot using a foil sampler. Afterward, the samples were mixed and homogenized and half of the remaining soil was passed through a <2 mm sieve to remove aboveground plant material, roots, and stones and stored at −40 °C prior to analysis. Soil DNA extraction was completed in 1 week, and the extracts were stored at −20 °C until further use. Soil enzyme activity was assayed in 2 weeks. A portion of each soil sample was air-dried, to measure physical and chemical properties. 

### 2.3. Measurements of Soil Physicochemical Property

Soil organic carbon (SOC) and total nitrogen (TN) content were determined on air dried, finely ground soil aliquots. Subsamples of 10 mg were weighted into tin caps and analyzed by a CN analyzer (TOC-L Analyzer and TN Unit, Shimadzu, Kyoto, Japan). 

### 2.4. Biological Analysis

#### 2.4.1. DNA Extraction and MiSeq Illumina Sequencing

Soil total genomic DNA was extracted from 0.5 g of soil sample using a PowerSoil Total DNA Isolation Kit (MoBio Labs, Solana Beach, CA, USA) according to the manufacturer’s instruction. DNA concentration and quality were checked using a NanoDrop spectrophotometer (NanoDrop Technologies Inc., Wilmington, DE, USA). Extracted DNA was diluted to 10 ng/μL and stored at −40 °C for downstream use. 

The primer pair 515F (5’-GTGCCAGCMGCCGCGG-3’) and reverse primer 907R (5’-CCGTCAATTCMTTTRAGTT-3’) with unique 6 nt barcode was used to amplify the hypervariable V4 region [19]. The sequencing samples were prepared using TruSeq DNA kit (Illumina, San Diago, CA, USA) according to manufacturer’s instructions. The purified library was diluted, denatured, re-diluted, mixed with PhiX (equal to 30% of final DNA amount) as described in the Illumina library preparation protocols, and then applied to a Miseq system (Illumina, San Diago, CA, USA) for sequencing with the Reagent Kit v2 2 × 250 bp (Illumina, San Diago, CA, USA) as described in the manufacturer’s manual.

#### 2.4.2. Sequencing Data Processing

Processing of the raw sequences obtained through Illumina sequencing was performed using the Quantitative Insights into Microbial Ecology (QIIME) pipeline (Knight Lab, Boulder, CT, USA) (QIIME: an pipeline for performing microbiome analysis). We assembled paired-end reads using FLASH. Reads with quality score lower than 20, ambiguous bases and improper primers were discarded before clustering. The resultant high quality sequences were then clustered into operational taxonomic units (OTUs) at 97% similarity using UPARSE algorithm. Simultaneously, chimeras were checked and eliminated during clustering. Taxonomic classification of representative sequences from individual OTU was performed by rdp classifier [20]. In order to compare relative difference between samples and retain the validity of most samples, a randomly selected subset of 7200 sequences per sample was performed for down-stream analyses. There were 1835534 high-quality sequences obtained, and sequence number of each sample was from 7599 to 101297 (Table S1).

#### 2.4.3. Soil Microbial Biomass 

The composited soils were divided into two parts: one was sieved to pass through a 2-mm mesh immediately and stored at 4 °C until analysis for the estimation of microbial biomass carbon (MBC) and microbial biomass nitrogen (MBN). Soil MBC and MBN were determined by the fumigation-extraction method [21,22]. The total organic carbon and nitrogen in the extracts were measured using a Multi N/C 3000 analyzer (Elementar Analysensysteme GmbH, Langenselbold, Germany).

MBC was calculated as:

MBC = EC/k_EC_ (1)

where EC = (organic C extracted from fumigated soils) − (organic C extracted from non-fumigated soils) and k_EC_ = 0.38 [23].

MBN was calculated as:

MBN= EN/k_EN_ (2)

where EN = (total N extracted from fumigated soils) − (total N extracted from non-fumigated soils) and k_EN_ = 0.45 [21].

### 2.5. Soil Enzymes 

*β*-Glucosidase enzyme activity was measured as described by [24]. The *β*-glucosidase enzyme was expressed as PNP μg/g, 1 h. Urease activity was measured using the modified [25]. The ammonium was measured spectrophotometrically at 578 nm, and the urease activity was expressed as N-NH_4_ μg/g, 1 h. Alkaline phosphatase activity was determined according to [26].The alkaline phosphatase was expressed as phenol μg/g, 1 h. The activity of catalase was determined by UV-Vis spectrophotometer (PGENERAL, Beijing, China) because the hydrogen peroxide had a strong light absorption at 240 nm. The catalase activity was calculated as the soilless control sample containing hydrogen peroxide minus the sum of the non-substrate control sample and the ordinary sample, and expressed as H_2_O_2_ μg/g, 1 h.

### 2.6. Statistical Analyses

The means and standard deviations of 3 replicates were calculated. The analysis of variance (ANOVA) was carried out using SPSS software package (version 13.0, IBM, New York, NY, USA). The significance of the parameters was tested using least significant difference (LSD) multiple range test at *p* < 0.05 or *p* < 0.01 after one-way ANOVA. The downstream analysis of soil DNA was performed using QIIME and R (Bell Laboratories, Murray Hill, NJ, USA) (R: A language and environment for statistical computing). Alpha diversity indices including the Shannon index and Chao1 index were calculated, and Bray-Curtis dissimilarity metrics were used for principle coordinate analysis (PCoA). Pearson correlation coefficients were calculated among soil basic chemical properties and enzyme activities. 

## 3. Results

### 3.1. Analysis of Microbial Community and Influencing Factors on Cultivated Land

#### 3.1.1. Alpha Diversity of Soil Microbial Community

Group O had the most observed operational taxonomic units (OTUs), followed by CK, N, and I (Table 2). O3 had the highest Shannon index of all the groups (*p* < 0.05), and I3 had the lowest (*p* < 0.05). There was a little difference in Simpson index among the set-asides, but I3 had the lowest (*p* < 0.01) (Table S2). I3 also displayed the lowest alpha diversity, in general. O3, O4 and O5 displayed significantly higher phylogenetic diversity (PD) whole tree values than did the other groups (*p* < 0.05), and I3 and N5 had significantly lower values compared with those of the other groups (*p* < 0.05). O3 and O5 displayed significantly higher Chao1 index values than did the other groups (*p* < 0.05), and N5 and I3 had significantly lower values compared with those of the other groups (*p* < 0.05). Among those groups, O3, O4 and O5 had significantly higher values than did the other groups (*p* < 0.05), and I3 had the lower values.

#### 3.1.2. Beta Diversity of Soil Microbial Community

**Figure 1:**
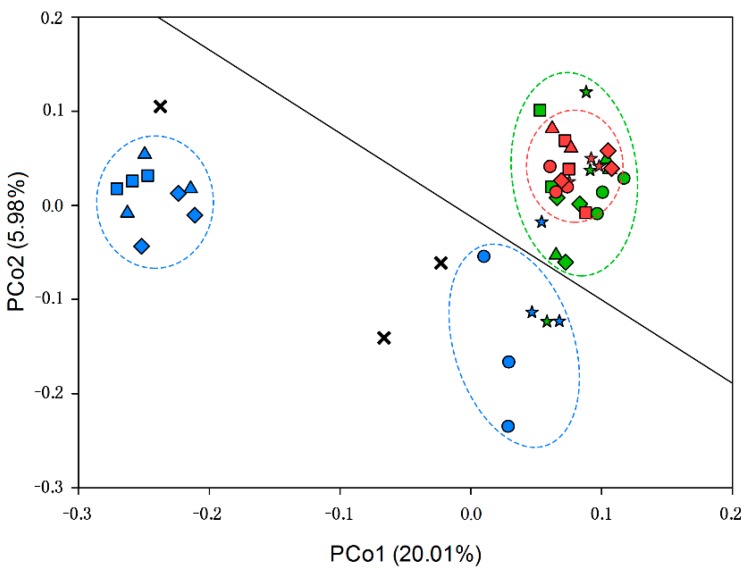
PCoA analysis of Bray-Curtis distance between samples. The points can be distinguished by color (management) and shape (plant types). Red indicated management group N; green represented group I; and blue indicated group O. Control group was indicated by black cross ”X”. Five-pointed star represented native plants; circular represented Soybean-Maize; square indicated Soybean-Elymus; triangle indicated Alfalfa-Maize; and diamond represented Alfalfa-Elymus. The black solid line, which divide the image into two parts, presented the fertilization status. The upper part of the black line indicated that no fertilizer was applied. In contrast, the lower part showed the application of fertilizer.

According to PCoA (Figure 1), CK was highly significantly different from the other groups (*p* < 0.01). Groups I and N were not significantly different, and intra-group differences were also not significant. First, O3, O4 and O5 did not significantly differ, but they did significantly differ from O1 and O2 (*p* < 0.01). Group O was significantly different from group N, except for O1 (*p* < 0.01). There was no significant difference between O1 and O2. The microbial community of CK exhibited a scattered distribution until the beginning of set-aside treatments (Figure 1).

**Figure 2:**
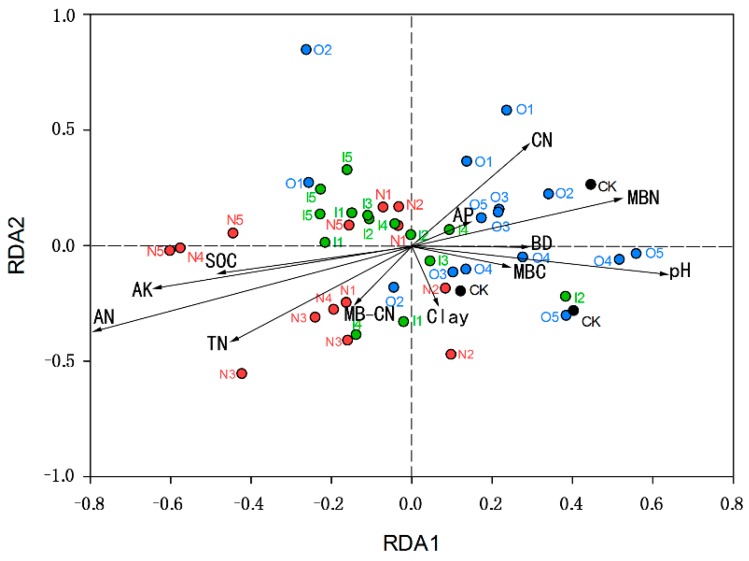
Redundancy analysis (RDA) of microbial communities using soil properties as environmental parameters. The different points color represents different management, which was consistent with Figure 1. The arrows represent soil properties, including carbon-nitrogen ratio (CN), available phosphorus (AP), microbial biomass nitrogen (MBN), bulk density (BD), microbial biomass carbon (MBC), microbial biomass carbon-nitrogen ratio (MB-CN), total nitrogen (TN), available nitrogen (AN), available potassium (AK), soil organic carbon (SOC). The circles were colored by managements, consistent with Figure 1.

According to the redundancy analysis (RDA) (Figure 2), the proportion explaining RDA1 was 21.97%; this proportion was greater than that for RDA2, which was 10.26%. On the RDA axes, the relationships between environmental variables and the axes were determined by projection length on the RDA axis (arrow length and angle size). 

Black spots represent the CK, distributed in the 1st and 4th quadrants. The N group is shown in red and mainly exists in the 2nd and 3rd quadrants (two points in the 1st and 4th quadrants). Group I is green and exists mainly in the 2nd and 3rd quadrants. Group O is blue and exists mainly in the 1st and 4th quadrants. The CK and group O are distributed on the right side of RDA2 axis, and N and I groups are mainly distributed on the left side of the RDA2 axis. The contents of AN, TN and AK were greater in the N group, and the group was influenced greatly. The O group had lower AN, TN and AK contents and was little impacted.

#### 3.1.3. Microbial Community Structure

**Figure 3:**
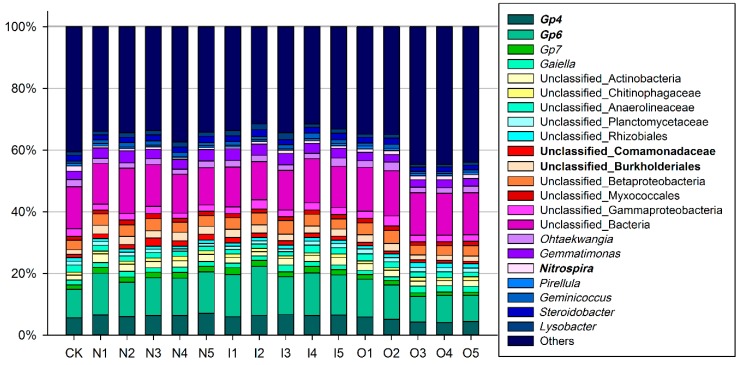
Relative abundance stacked bar plot of the microbial genera. The samples were summarized by management-plant types.

A total of 457 genera were identified from these samples and used to study bacterial community structure. Figure 3 shows that set-aside treatments transformed the bacterial community structure. Many bacterial genera with greater relative percentages, such as *Gp4* (4.16–7.10%), *Gp6* (8.26–15.91%), *Nitrospira* (0.47–1.67%), unclassified Comamonadaceae (0.80–2.61%), unclassified Burkholderiales (1.50–2.81%) and unclassified bacteria (10.67–14.73%), were identified in farmland soils. *Nitrospira* in CK was significantly higher than in groups N and I, except I3 (*p* < 0.05), and group N had significantly less *Nitrospira* than did group O (*p* < 0.05). *Nitrospira* of I2 and I3 did not significantly different from that of O2 and O3. The correlation of *Gp6* and unclassified Burkholderiales was highly significant (r = 0.740, *p* < 0.001); at the same time, *Gp6* and unclassified Burkholderiales were also significantly negatively correlated with *Nitrospira* (*p* < 0.001). CK, O3, O4 and O5 displayed significantly less Gp6 and unclassified Burkholderiales than did O1 and O2 (*p* < 0.05).

### 3.2. Soil Microbial Biomass Carbon and Nitrogen 

The SOC among set-asides did not significantly differ (Table 3). The TN was highest in N2 (*p* < 0.05). The carbon/nitrogen ratio (C/N) of N2 (5.93) was the highest (*p* < 0.05); I5 was significantly higher than both O5 (*p* < 0.05) and CK. Group O had slightly higher MBC than did groups I and N; I1 had significantly less than O5 and N1 (*p* < 0.05). The microbial biomass C/N was not consistent, but differences were not significant. CK had a higher proportion of MBC than did N2, N4, N5, I1 to I3, and O1, and the proportion in N1 (4.20%) was markedly higher than that in I1 and O1 (2.76% and 3.16%, respectively). The effects of fertilizer on the proportion of MBN were more significant.

### 3.3. Soil Enzyme

**Figure 4:**
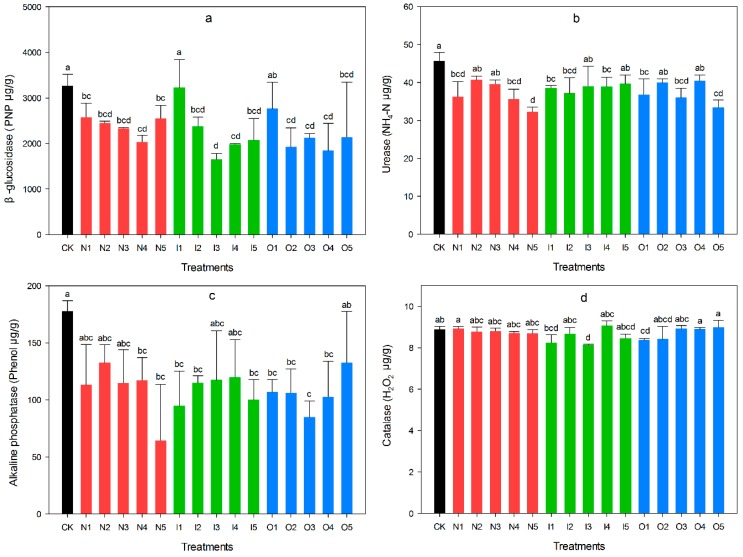
Changes of four enzyme activities in different set-aside patterns in spring, (**a**) *β*-glucosidase (**b**) urease (**c**) alkaline phosphatase (**d**) catalase. The black bar represented control (CK), red bar represented no management (N1–N5), the green bar represented only irrigation (I1–I5), the blue bar represented organic management (O1–O5).

The β-glucosidase activity is shown in Figure 4a. CK and I1 presented significantly higher than did the others, except O1, whereas I3 presented significantly lower values than did CK, I1 and O1 (*p* < 0.05). AP was significantly correlated with β-glucosidase activity (r = 0.451, *p* < 0.01) (Table 4). CK had the highest urease activity, and N5 had the lowest (*p* < 0.05, Figure 4b), and urease was significantly correlated with AN (r = 0.451, *p* < 0.01). O3 had significantly lower alkaline phosphatase activity than did CK and O5 (*p* < 0.05) (Figure 4c), and alkaline phosphatase was significantly correlated with AN (r = 0.535, *p* < 0.01). Catalase activity is shown in Figure 4d. N1, O4 and O5 were significantly higher activity than did I1, I3 and O1 (*p* < 0.05), and the activity was highly significantly correlated with MBC (r = 0.775, *p* < 0.01).

## 4. Discussion 

Management regimes showed more significant effects than did vegetation in short-term set-asides. Organic manure and chemical fertilizer clearly promoted the soil microbial alpha diversity of soil communities among plots. However, the organic manure was more effective than chemical fertilizer, because external nutrient resources and bacterial inputs altered intrinsic soil microbial communities [27].

No management presented more clustering than irrigation in terms of soil beta diversity. Manure seemed to cause clustering, with the exceptions of native plants and maize-soybean, possibly because those plants were less influenced by organic manure. According to the RDA, the microbial communities were susceptible to pH, AN, TN, MBN and SOC. Considering the high local soil pH, we suppose that the change in microorganisms is related to native soil conditions. In the absence of fertilizer, soil organic matter and nitrogen deficiency become important factors restricting soil microbial communities at this stage [28].

There were 23 major genera that changed in the soil (the relative abundance must be >0.4%), which represented significant changes. *Nitrospira* responds positively to high nitrogen availability and high nitrite oxidation potential [29]. In the short term, nitrification decreased in the set-aside areas because of fertilizer deficiency, and organic manure could relieve this reduction. This phenomenon could explain the loss of ammonium nitrogen in set-aside lands. However, *Gp6* which is the predominate subgroup belonging to Acidobacteria, showed opposite results, in that the abundance decreased with fertilizer use. According to the previous research, Acidobacteria *Gp4* and *Gp6* are more abundant in soils with relatively high SOC [30]. Our results confirm the relationships between *Gp4*, *Gp6* and carbon, and the relationships were all opposite those with *Nitrospira* (*p* < 0.01). The unclassified Burkholderiales and unclassified Comamonadaceae may contribute to denitrification in farmlands; this phenomenon may be related to the biological control of natural soil diseases and could potentially promote plant growth [31,32]. Similarly, unclassified Burkholderiales and unclassified Comamonadaceae all showed opposite correlations with *Nitrospira* (*p* < 0.05). Therefore, manure can improve the nitrification but reduces both carbon aggregation and soil disease resistance. After multiple comparison tests, O1, I2, I4 and I5 were relatively more balanced than were the others, especially I5. We therefore suggest that only the irrigation management regime can maintain the short-term balance of soil microbial functions.

The differences of SOC were not significant so manure didn’t show obvious effects during the stage [33]. Unmanaged maize-soybean resulted in relatively lower C/N values compared with those of other management regimes. Therefore, this is consistent with previous research that abundant aboveground vegetation could cause increased nitrogen consumption [34]. Native plants displayed significantly lower MBC under only irrigation but relatively high MBC under no management, whereas MBC was modest under organic management. The microbial biomass C/N ratio could reflect the proportion of fungi such that no management regime contributed to fungi in soil [35]. When CK was set as the comparison, the microbial biomass C/N ratio indicates N1–N4, I1, I4, O2 and O5 were becoming dominated by fungi, and the others were becoming dominated by bacteria. The ratio of MBC to SOC could then be used to monitor changes in soil organic matter, and the normal range is 1–5% [36]. Here, we also set CK as the critical value. Based on the above results, we could obtain the following major types:

(i). Fungal dominant with more organic matter, which occurred in N2, I1 and I4.
(ii). Fungal dominant with less organic matter, which occurred in N1, N3, N4, O2 and O5.
(iii). Bacterial dominant with more organic matter, which occurred in N5, I2, I3 and O1.
(iv). Bacterial dominant with less organic matter, which occurred in I5, O3 and O4.

Considering that increased fungal efficiency is unsubstantiated, some studies have supported a greater carbon storage and slower carbon turnover [37,38]. We believe (iv) is necessary to enhance organic matter via manure supplementation. N1 and O5 exceeded normal levels (1–5%), which may indicate the risk of organic matter deficiency. Therefore, organic matter levels should be monitored in the future. According to the current study, bacterial-dominated food webs do not cycle faster or replenish more carbon and nitrogen than do fungal-dominated food webs [39]. For this reason, we conservatively argue that (ii) and (iv) need organic matter supplementation. The addition of farmyard manure unexpectedly led to a lack of organic matter, except in the native plant treatment. When taking microbial communities together, our results confirm that O1, I2 and I4 are relatively stable set-aside patterns. It worth noting that group O didn’t show any significant advantage over CK, which was also noted in some previous studies [40,41]. The major reason is no-tillage, the tillage method of our research, which encouraged the emergence of some weeds [42]. The boosting of vegetation may increase the deficiency of soil nutrients, which led to a decrease in microbial biomass and diversity [43]. Though the advantage of manure was not significant in the short-term, it still provided enough nutrients and changed soil microbiota which shouldn’t be ignored in the set-aside. Mucoraceous fungi are the source of *β*-glucosidase [44]. Native plants were the best under the irrigation and organic management regimes. Although the accumulation of fungal pathogens could be caused by continuous sorghum cropping [45], the promotion of *β*-glucosidase generated by sorghum should not be ignored. The addition of leguminous vegetation weakened the advantage of sorghum and decreased the short-term activity of *β*-glucosidase. 

Urease activity often indicates organic nitrogen mineralization and reflects the soil microbial processes and nitrogen supply capacity [46]. N5 and O5 showed significantly lower urease activity than did the other groups, confirming that the activity in alfalfa-dahurian wild rye was stimulated by irrigation. According to the empirical data, the plants of O5 presented lush growth; therefore, those plants appear to require more water and manure. Although urease is an important indicator of soil fertility, this enzyme converts organic nitrogen into ammonium nitrogen, and the rapid volatilization of ammonium nitrogen reduces nutrient components, resulting in the inefficient degradation of fertilizers [47].

According to the alkaline phosphatase results, tillage significantly influenced alkaline phosphatase activity, and no-till management promoted this activity [48]. However, we observed that conventional farming could also maintain a high level of alkaline phosphatase activity over time. Catalase presented highly significant positive correlations with microbial biomass. Catalase activity of I3 was significantly lower than that of group N; no other significant differences were observed in group I. A similar situation occurred for microbial alpha diversity.

Overall, set-asides did not benefit soil enzymes remarkably. Nevertheless, set-asides significantly affected microbial biomass and microbial communities. Farmyard manure likely led increased consumption of soil organic matter and enhanced nitrification, which were opposite effects to those observed under no management. However, some set-asides appeared more stable, including O1, I2 and I4. According to a cost-benefit analysis, soybean-maize and alfalfa-maize treatments with irrigation were the most economical and worth adopting. In other words, the use of native plants with farmyard manure is recommended for the most rapid improvement of arable land quality.

## 5. Conclusions 

Our study has provided a new perspective of research on set-aside treatments in China, although set-asides were used for short period. The results did not show obvious benefits to soil enzymes in the short-term, but short-term set-asides could affect soil microbial functions via the microbial biomass and communities. At the same time, some set-asides showed more reliability. The selection of set-aside patterns (long or short term) should consider farmland background, and the deep understanding of set-aside management is important.

## Figures and Tables

**Table 1:** Treatments applied for set-aside.

Vegetation	Management Groups			
	No Management (N)	Irrigation (I)	Organic Manure and Irrigation (O)	Control Group (CK)
Native plants (1)	N1	I1	O1	n
Soybean-maize (2)	N2	I2	O2	n
Soybean-dahurian wild rye (3)	N3	I3	O3	n
Alfalfa-maize (4)	N4	I4	O4	n
Alfalfa-dahurian wild rye (5)	N5	I5	O5	n
Control group (CK)	n	n	n	CK

The n represented no relationship with other managements.

**Table 2:** Alpha diversity of soil microbial community.

Sample ID	Shannon Index	Observed_Otus	Chao1 Index	PD_Whole_Tree
CK	9.88 ± 0.07*_abc_*	1986.00 ± 25.96*_abc_*	3309.87 ± 40.61*_abc_*	111.25 ± 2.11*_abc_*
N1	9.73 ± 0.03*_bcd_*	1887.33 ± 9.03*_cdef_*	3201.78 ± 132.00*_abcd_*	105.46 ± 0.80*_bcd_*
N2	9.85 ± 0.08*_abc_*	1941.33 ± 53.32*_bcd_*	3205.43 ± 158.09*_abcd_*	106.96 ± 2.84*_bcd_*
N3	9.73 ± 0.09*_bcd_*	1929.67 ± 41.49*_bcd_*	3200.35 ± 113.72*_abcd_*	106.74 ± 3.00*_bcd_*
N4	9.76 ± 0.04*_abcd_*	1858.67 ± 38.42*_def_*	3051.08 ± 78.06*_cde_*	101.33 ± 2.12*_df_*
N5	9.53 ± 0.15*_d_*	1773.50 ± 97.50*_fg_*	2871.64 ± 141.33*_e_*	97.50 ± 5.01*_f_*
I1	9.61 ± 0.07*_cd_*	1801.00 ± 42.62*_efg_*	2986.73 ± 118.40*_de_*	101.16 ± 3.10*_df_*
I2	9.55 ± 0.05*_d_*	1803.67 ± 46.95*_efg_*	3056.10 ± 160.58*_cde_*	101.38 ± 3.14*_df_*
I3	9.23 ± 0.43*_e_*	1714.50 ± 139.50*_g_*	2866.69 ± 315.44*_e_*	96.85 ± 6.06*_f_*
I4	9.73 ± 0.10*_bcd_*	1896.67 ± 60.81*_bcde_*	3110.23 ± 131.15*_bcde_*	104.83 ± 3.54*_cd_*
I5	9.73 ± 0.07*_bcd_*	1899.00 ± 22.55*_bcde_*	3149.99 ± 101.43*_abcde_*	104.73 ± 2.43*_cd_*
O1	9.87 ± 0.04*_abc_*	2016.33 ± 23.70*_ab_*	3271.84 ± 59.80*_abcd_*	112.05 ± 1.11*_ab_*
O2	9.89 ± 0.04*_ab_*	2001.33 ± 43.76*_abc_*	3330.37 ± 129.47*_abc_*	111.52 ± 2.65*_ab_*
O3	10.02 ± 0.03*_a_*	2088.67 ± 20.76*_a_*	3463.42 ± 13.08*_a_*	116.00 ± 0.83*_a_*
O4	9.98 ± 0.03*_ab_*	2062.67 ± 17.46*_a_*	3412.02 ± 53.47*_ab_*	114.04 ± 1.03*_a_*
O5	9.10 ± 0.02*_ab_*	2073.67 ± 27.40*_a_*	3440.88 ± 110.25*_a_*	115.26 ± 0.60*_a_*

Arrange letters a, b, c, d, e and f from large to small based on average values. Values in the same column followed by the same letter are not different (*p* > 0.05) according to a General Linear Model (GLM) protected least significant difference (LSD) test.

**Table 3:** The change of soil carbon and nitrogen of arable lands in spring.

Sample ID	SOC	Soil TN	C-to-N Ratio of Soil	MBC	MBN	MBC-to-MBN Ratio	% of Total C or N as	
	(g/kg)			(mg/kg)			MBC	MBN
CK	5.74	0.80*_b_*	7.17*_abc_*	256.48*_abc_*	20.51*_ab_*	12.56	4.47	3.61
N1	5.50	0.90*_b_*	6.14*_abc_*	293.86*_ab_*	20.88*_ab_*	14.34	5.34	3.67
N2	5.89	1.36*_a_*	4.64*_c_*	249.82*_abc_*	18.60*_ab_*	13.67	4.24	2.80
N3	5.28	0.95*_b_*	5.53*_bc_*	256.30*_abc_*	17.60*_b_*	14.55	4.85	2.50
N4	5.32	1.00*_b_*	5.43*_bc_*	241.32*_abc_*	16.43*_b_*	14.86	4.54	2.09
N5	5.23	0.86*_b_*	6.08*_abc_*	226.64*_bc_*	21.19*_ab_*	11.21	4.33	3.30
I1	6.01	0.78*_b_*	7.74*_ab_*	219.12*_c_*	21.02*_ab_*	12.66	3.65	3.10
I2	6.36	0.96*_b_*	6.63*_abc_*	268.66*_abc_*	22.37*_ab_*	12.27	4.22	2.98
I3	5.92	0.92*_b_*	6.43*_abc_*	225.88*_bc_*	20.22*_ab_*	11.37	3.82	3.05
I4	6.02	0.86*_b_*	7.01*_abc_*	268.93*_abc_*	19.10*_ab_*	14.33	4.47	3.09
I5	5.29	0.71*_b_*	7.61*_ab_*	255.33*_abc_*	20.84*_ab_*	12.30	4.83	3.67
O1	6.22	0.73*_b_*	8.47*_a_*	235.93*_abc_*	21.26*_ab_*	11.13	3.79	3.73
O2	5.19	0.71*_b_*	7.37*_ab_*	255.92*_abc_*	20.18*_ab_*	12.84	4.93	3.87
O3	5.37	0.81*_b_*	6.79*_abc_*	253.21*_abc_*	22.23*_ab_*	12.21	4.72	3.33
O4	5.66	0.80*_b_*	7.00*_abc_*	278.92*_abc_*	24.75*_a_*	11.38	4.93	4.28
O5	5.63	0.90*_b_*	6.32*_abc_*	303.56*_a_*	21.53*_ab_*	14.43	5.39	3.36

Arrange letters a, b and c from large to small based on average values. Values in the same column followed by the same letter are not different (*p* > 0.05) according to a GLM protected LSD test. SOC: soil organic carbon; MBC: microbial biomass carbon; MBN: microbial biomass nitrogen.

**Table 4:** The Pearson correlation coefficients between soil properties and enzyme activities.

Soil Properties	pH	AN	AK	MBC	SOC	TN	AP	APL	β	Ur	CAT
pH	1.000										
AN	−0.302 *	1.000									
AK	−0.178	0.243	1.000								
MBC	0.034	0.057	0.000	1.000							
SOC	−0.215	0.386 **	0.396 **	−0.091	1.000						
TN	−0.155	0.620 **	0.321 *	0.049	0.358 *	1.000					
AP	−0.333 *	0.285 *	−0.051	−0.044	0.136	−0.128	1.000				
APL ^§^	−0.256	0.535 **	0.237	0.242	0.343*	0.303 *	0.263	1.000			
*β*^§^	−0.131	0.179	0.013	−0.071	0.023	−0.047	0.451 **	0.183	1.000		
Ur ^§^	−0.330 *	0.372 **	0.200	0.055	0.221	0.185	0.548 **	0.422 **	0.116	1.000	
CAT ^§^	−0.071	0.172	−0.042	0.775 **	−0.111	0.172	−0.069	0.226	0.029	−0.032	1.000

* *p* = 0.05 (significant); ** *p* = 0.01 (significant); ^§^ indicates the abbreviation for soil enzyme; ALP: alkaline phosphatase; *β: β*-glucosidase; Ur: urease; CAT: catalase.

## Supplementary Materials

The following are available online at www.mdpi.com/1660-4601/14/8/913/s1, Table S1: Summary of samples’ sequence counts, Table S2: Simpson index of soil microbial community.
