# Supplementary material for: Effects of Short-Term Set-Aside Management Practices on Soil Microorganism and Enzyme Activity in China
Source: Int J Environ Res Public Health. 2017 Aug 14;14(8):913. doi: 10.3390/ijerph14080913 (PMC5580616; doi:10.3390/ijerph14080913)
Supplement: Supplementary file 1 [file ijerph-14-00913-s001.pdf]

Table S1. Summary of samples' sequence counts.

Sample ID	Sequence Counts
N1	30434
N1	7599
N1	34279
N2	57066
N2	24690
N2	17511
N3	26311
N3	41523
N3	22255
N4 §	4028
N4	25507
N4	19962
N5	49211
N5	18085
N5	23328
I1	38160
I1	29748
I1	45994
I2	36883
I2	41125
I2	8807
I3	60784
I3§	718
I3	53642
I4	15450
I4	17912
I4	16768
I5	53622
I5	41240
I5	61650
O1	14699
O1	42747
O1	10538
O2	54867
O2	45563
O2	51381

O3	57264
O3	37119
O3	55375
O4	39647
O4	90195
O4	8165
O5	75831
O5	80873
O5	8539
CK	101297
CK	81868
CK	60020
Sum	1835534
Mean	39902.9130434783

The § indicated samples which were abandoned because of insufficient sequences based on 7200 sequences demand.

Table S2. Simpson index of soil microbial community.

**Treatments**	**Simpson index**
CK	0.997±0.0002*abc*
N1	0.996±0.0001*abcde*
N2	0.997±0.0003*abcd*
N3	0.996±0.0003*abcde*
N4	0.997±0.0001*abcde*
N5	0.996±0.0008*cde*
I1	0.996±0.0007*de*
I2	0.996±0.0006*e*
I3	0.994±0.003*f*
I4	0.996±0.0003*bcde*
I5	0.996±0.0004*cde*
O1	0.997±0.0001*abcde*
O2	0.997±0.0001*abc*
O3	0.998±0.0002*a*
O4	0.997±0.0002*abc*
O5	0.998±0.0000*ab*

Arrange letters a, b, c, d, e and f from large to small based on average values. Values in the same column followed by the same letter are not different ($P > 0.05$) according to a GLM protected LSD test.
